# Update of the *Anopheles gambiae *PEST genome assembly

**DOI:** 10.1186/gb-2007-8-1-r5

**Published:** 2007-01-08

**Authors:** Maria V Sharakhova, Martin P Hammond, Neil F Lobo, Jaroslaw Krzywinski, Maria F Unger, Maureen E Hillenmeyer, Robert V Bruggner, Ewan Birney, Frank H Collins

**Affiliations:** 1 Center for Global Health and Infectious Diseases, University of Notre Dame, Galvin Life Sciences Building, Notre Dame, IN 46556-0369, USA; 2 Department of Entomology, College of Agriculture and Life Science, Virginia Polytechnic Institute and State University, Blacksburg, VA 24061-0319, USA; 3 European Bioinformatics Institute (EMBL-EBI), Wellcome Trust Genome Campus, Hinxton, Cambridge, CB10 1SD, UK; 4 Department of Biology, University of Texas at Arlington, Arlington, TX 76019, USA; 5 School of Medicine - IDP - Biomedical Informatics, Stanford University, Stanford, CA 94305, USA

## Abstract

**Background:**

The genome of *Anopheles gambiae*, the major vector of malaria, was sequenced and assembled in 2002. This initial genome assembly and analysis made available to the scientific community was complicated by the presence of assembly issues, such as scaffolds with no chromosomal location, no sequence data for the Y chromosome, haplotype polymorphisms resulting in two different genome assemblies in limited regions and contaminating bacterial DNA.

**Results:**

Polytene chromosome *in situ *hybridization with cDNA clones was used to place 15 unmapped scaffolds (sizes totaling 5.34 Mbp) in the pericentromeric regions of the chromosomes and oriented a further 9 scaffolds. Additional analysis by *in situ *hybridization of bacterial artificial chromosome (BAC) clones placed 1.32 Mbp (5 scaffolds) in the physical gaps between scaffolds on euchromatic parts of the chromosomes. The Y chromosome sequence information (0.18 Mbp) remains highly incomplete and fragmented among 55 short scaffolds. Analysis of BAC end sequences showed that 22 inter-scaffold gaps were spanned by BAC clones. Unmapped scaffolds were also aligned to the chromosome assemblies *in silico*, identifying regions totaling 8.18 Mbp (144 scaffolds) that are probably represented in the genome project by two alternative assemblies. An additional 3.53 Mbp of alternative assembly was identified within mapped scaffolds. Scaffolds comprising 1.97 Mbp (679 small scaffolds) were identified as probably derived from contaminating bacterial DNA. In total, about 33% of previously unmapped sequences were placed on the chromosomes.

**Conclusion:**

This study has used new approaches to improve the physical map and assembly of the *A. gambiae *genome.

## Abstract 2

An update on the *Anopheles *gambiae PEST genome assembly places about 33% of previously unmapped sequences on the chromosomes.

## Background

The genome of *Anopheles gambiae*, the major vector of malaria in Africa, was sequenced by a whole-genome shotgun approach [1]. Physical mapping of the genome was conducted by *in situ *hybridization of about 2,000 bacterial artificial chromosome (BAC) clones on ovarian nurse cell polytene chromosomes. As a result, in the first publication of the *A. gambiae *physical map, 67 scaffolds equivalent to 227 mega-base-pairs (Mbp) were assigned to chromosomes. Of these, 52 scaffolds were oriented. However, approximately 18% of the assembled *A. gambiae *genome was represented in scaffolds that did not have a chromosomal location assigned. About 50 Mbp in the assembly were assigned with arbitrary order and orientation to an unmapped chromosome [2]. In this study, new approaches were used to improve the physical map and assembly of the *A. gambiae *genome.

The most poorly mapped parts of the *A. gambiae *genome were the pericentromeric regions of the chromosomes. These chromosomal regions are made up of highly and moderately repetitive DNA sequences [3,4] that are extremely depleted of genes [5] and form specific heterochromatic structures on chromosomes [6,7]. Pericentromeric heterochromatin plays an important role in many biological processes, such as cell division [8], meiotic pairing [9], regulation of DNA replication and gene expression [10,11], and is generally associated with gene silencing [12,13]. However, the assembly and physical mapping of these regions is a difficult part of any genome project [14-19]. In *Drosophila melanogaster*, for example, one-third of the 180 Mbp genome is centric heterochromatin; but in the first genome publication only 2% of the sequence reads contained heterochromatic simple sequence repeats [20], and only 3 scaffolds corresponding to 3.8 Mb were mapped in centromeric areas [14].

According to Cot analysis, 33% (about 86 Mbp) of the *A. gambiae *genome corresponds to repetitive elements [21]. The highest density of repeats is located in pericentromeric regions and forms the completely heterochromatic Y chromosome [22]. In contrast with *Drosophila*, short simple repeats are not expanded in the *A. gambiae *genome; therefore, cloning of the heterochromatic portion of the genome was more successful. However, in the first publication of the *A. gambiae *genome only 9 scaffolds, with a total size of 3.3 Mbp, were mapped to pericentromeric regions on chromosomes [1]. Mapping is difficult because BAC clones representing pericentromeric regions are likely to map to multiple locations due to their high repeat content. In previous work 27 BAC clones hybridized to all centromeric regions on the chromosomes and 116 BAC clones hybridized to pericentromeric regions and multiple locations on the chromosomes [1]. To determine the genomic location of heterochromatic scaffolds, cDNA clones from the Normalized Anopheles Pool (ANGNAP1) library with sequences matching regions of these scaffolds were mapped to the chromosomes. Additionally, this approach was used to orient scaffolds that were not previously oriented. For some scaffolds, PCR amplified DNA of genes predicted in the scaffolds was used for physical mapping.

No sequence data were assigned to the Y chromosome in the original publication of the *A. gambiae *genome [1]. Subsequent studies revealed numerous repetitive sequences on the Y chromosome, including four families of satellite DNA and a massive accumulation of several transposable elements, consistent with the fully heterochromatic nature of that chromosome [23,24]. Only one Y-specific scaffold contained an open reading frame that appeared to correspond to a gene fragment and was expressed exclusively in males. However, a recent extensive bioinformatics-based search failed to reveal other Y-linked scaffolds containing gene sequences [25].

Another significant problem in the *A. gambiae *genome assembly was the existence of 64 physical gaps between the mapped scaffolds [1]. BAC and cDNA clones were used for *in situ *hybridization to physically assign 5 scaffolds with a total size of 1.3 Mbp in these gaps. In addition, systematic *in silico *analysis of BAC end sequences (BESs) from the ND-1 [1] and ND-TAM [26] BAC libraries identified BAC clones that span a third of the physical gaps. The sequencing of these clones would allow further improvement of the *A. gambiae *genome assembly.

Genetic variation within the *A. gambiae *genome posed another challenge for mapping and assembly [1]. *A. gambiae *is a highly polymorphic species, characterized by the presence of five chromosomal forms (Bamako, Mopti, Savanna, Bissau and Forest); sympatric populations of the Bamako, Mopti and Savanna forms are at least partially isolated from each other in Mali. These chromosomal forms can be identified by paracentric inversions of the 2R chromosomal arm and have different adaptation to certain climatic conditions and human environments [27-29]. Moreover, an additional type of polymorphism, termed M and S molecular forms, has been revealed in natural populations of *A. gambiae *by differences in ribosomal DNA [30,31]. The PEST strain, selected for the genome sequencing because it had the standard chromosomal arrangement, was produced by crossing a laboratory strain originating in Nigeria with the offspring of field-collected *A. gambiae *from western Kenya [1]. As a result of the high level of polymorphism within the strain, some regions of the genome appeared as two different assemblies ('haplotypes') within the set of scaffolds. Holt *et al*. [1] estimated that the presence of alternative assemblies led to overrepresentation of the size of the genome by about 21.3 Mbp. Additional analysis of the scaffold sequences *in silico *identified 144 previously unplaced scaffolds totaling 8.18 Mbp that are probable alternative assemblies of regions already placed on the chromosomes. In addition, 20 cases totaling 3.53 Mbp of sequence were identified where the adjacent ends of large mapped scaffolds appear to be alternative assemblies of the same region.

The genomic libraries used for the sequencing of the *A. gambiae *genome were contaminated by bacterial DNA [1]. By bioinformatics approaches, 679 scaffolds with a total size of 1.97 Mbp were determined to be derived from contaminating bacterial DNA.

## Results

The revised *A. gambiae *PEST assembly is available at GenBank. The scaffold entries have information about alternative assembly regions and all other corresponding information. The new RefSeq entries reflect the revised chromosome assemblies (GenBank: CM000356-CM000360).

### Physical mapping and scaffold orientation in the pericentromeric regions

**Figure 1:**
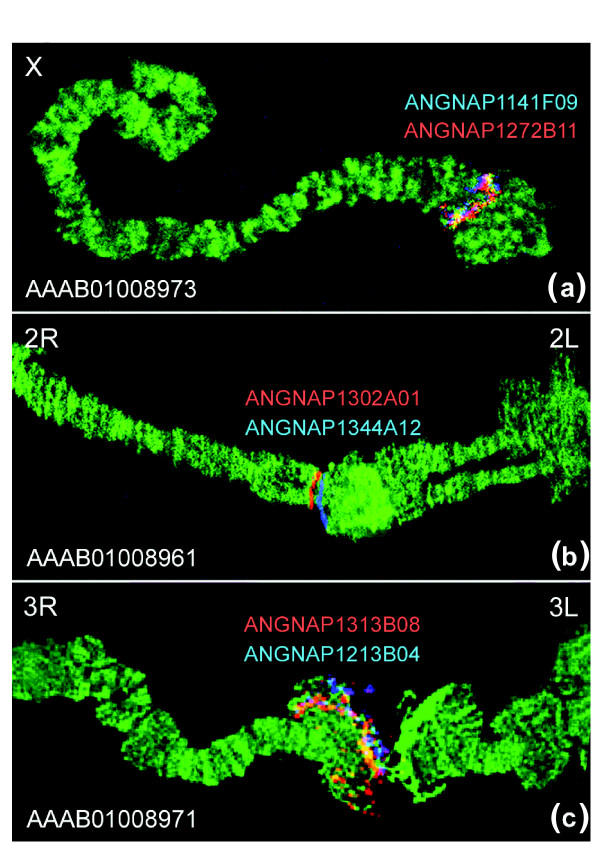
Results of *in situ *hybridization of cDNA clones to the heterochromatic regions on the polytene chromosomes of *A. gambiae*. Two cDNA clones were labeled with red Cy3 and blue Cy5 dyes and hybridized to the polytene chromosomes: the red signals indicate the beginning and the blue signals show the end of the scaffolds. The location of the scaffolds, **(a) **AAAB01008973, **(b) **AAAB01008961 and **(c) **AAAB01008971, were indicated by *in situ *hybridization of the cDNA clones: ANP1272B11, ANP1141F09 (a); ANP1302A01, ANP1344A01 (b) and ANP131B08, ANP121D04 (c) on the chromosome X (a), 2 (b) and 3 (c).

Pericentromeric regions are probably under-represented in the genome assembly because the scaffolds from these regions, although assembled, cannot be localized on the chromosome. The likely reason they fail to localize correctly is that they contain a large percentage of highly repeated sequences, so large probes such as the BAC clones previously used to map the scaffolds give ambiguous results - hybridization to multiple regions. This was overcome by using unique sequences in the scaffolds as probes for *in situ *hybridization on the ovarian polytene chromosomes. A good source of unique sequences is cDNAs from unique genes encoded in the scaffolds. To detect the genes in the unassigned scaffolds, cDNA sequences from the ANGNAP1 library were compared to the scaffold sequences. Clones representing unique sequences near the ends of the scaffolds were selected for use as probes for *in situ *hybridization. To differentiate scaffold ends, cDNAs from opposite ends were labeled with red Cy3 and blue Cy5 dyes. Typical results from *in situ *hybridizations using this technique are shown in Figure 1. The results from numerous hybridization experiments demonstrated that the scaffolds could generally be oriented on the polytene chromosome when the labeled target sequences were located more than 100 kb apart on the scaffold. In some cases, cDNAs were not available to represent the unique sequences at the end of a scaffold; in those cases, probes were made by PCR amplification of unique sequence from BAC clones. Although the use of PCR amplified genes was less successful than the use of cDNA sequences, three scaffolds (AAAB01008973, AAAB01008949 and AAAB01008942) were positioned by this technique.

#### Chromosome X

**Figure 2:**
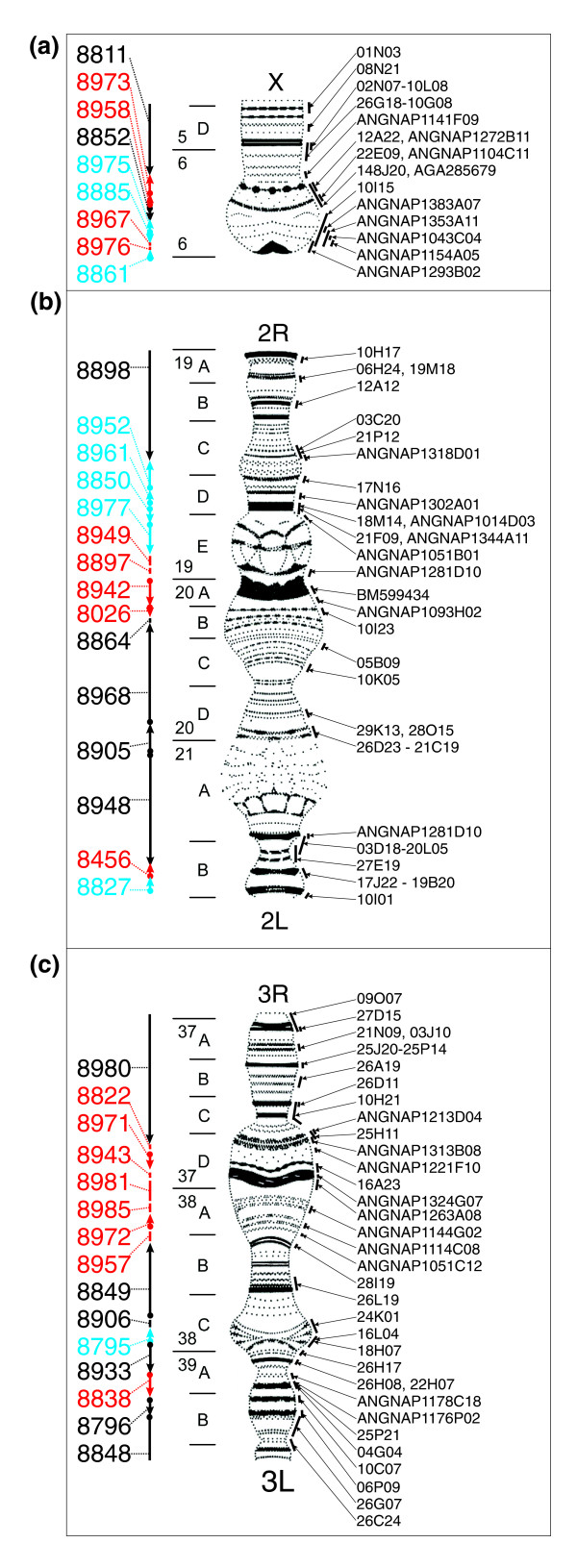
Scaffolds located in pericentromeric regions on *A. gambiae *chromosomes. Black and red lines and arrows on the left side of the picture correspond to the scaffolds previously and newly mapped to the pericentromeric regions of chromosomes **(a) **X, **(b) **2 and **(c) **3, respectively; blue arrows indicate newly oriented scaffolds. The dots on the arrows show the beginning of the scaffolds and the arrowheads correspond to the end of the scaffolds. The scaffolds are identified by the last four digits of the scaffold ID. The scale on the left side of the chromosomes indicates divisions and subdivisions in these regions. Black arrows on the right side of the picture show the location of the PCR amplified gene-fragments and BAC and cDNA clones.

By cDNA and PCR fragment physical mapping, four scaffolds with lengths between 400 and 600 kbp were placed in the pericentromeric region on chromosome X (Additional data file 1). Scaffolds AAAB01008973 and AAAB01008858 were localized and oriented to the distal part of the X chromosome pericentromeric region 6 (Figure 2a). Scaffolds AAAB01008967 and AAAB01008976 were mapped more proximally to the pericentromeric region (Figure 2a). Orientation of these scaffolds was not possible because cDNA clones within the scaffolds hybridized to the same places on the chromosome. By the same method, previously mapped but unoriented scaffolds AAAB01008975 and AAAB01008885 were oriented (Figure 2a). Scaffold AAAB01008861 was oriented and mapped more precisely as the most proximal scaffold on the X chromosome (Figure 2a). Two cDNA clones from that scaffold were localized to the most proximal part of the pericentomeric heterochromatin. cDNA clone ANGNAP1293B02, which hybridized to the condensed heterochromatic band on the X chromosome, also labeled nucleoli in all cells on the slide. BLASTN analysis demonstrated significant similarity of this cDNA to ribosomal RNA genes of *A. albimanus *and *Aedes albopictus*. These genes are not currently annotated in the *A. gambiae *genome. This area of the X chromosome has a significant number of gaps, which may have hindered *in silico *annotation. BLASTN analysis also showed localization of *A. gambiae *ribosomal RNA genes in scaffold AAAB01008976, which is the adjacent mapped scaffold. Thus, this area can be described as a nucleolar-organizing region for the polytene X chromosome.

#### Chromosome 2

Four scaffolds were mapped to the pericentromeric region of chromosome 2 (Additional data file 1). Two scaffolds AAAB01008949 and AAAB01008897 were placed on the right arm (2R) of this chromosome (Figure 2b). Scaffold AAAB01008942 was assigned to the very proximal end of the 2L arm (Figure 2b), and scaffold AAAB01008026 was mapped on the distal part of region 20A (Figure 2b). Both scaffolds mapped to the 2L arm have been oriented. The distal boundary of scaffold AAAB01008987 was also mapped to the telomeric region of the 2R arm. The last BAC clone, 170B21, from this scaffold hybridized to the pair of distal dark bands in subdivision 7A. Additional analysis of previously mapped BAC clones showed that the telomeric end of the 2L chromosomal arm was covered by scaffold AAAB01008807.

#### Chromosome 3

Eight scaffolds were assigned to the pericentromeric region of chromosome 3 (Additional data file 1; Figure 2c). Scaffold AAAB01008822 was localized on the distal part of region 37D on the 3R arm. Scaffold AAAB01008943 covered the proximal part of this region and reached the centromeric block of 3R. Scaffolds AAAB01008957, AAAB01008972, AAAB01008985 and AAAB01008981 have been placed in region 38A of the 3L arm. Only two of these scaffolds, AAAB01008943 and AAAB01008972, have been oriented. In addition, scaffold AAAB01008795 was oriented in the region 38C, and the distal boundary of the scaffold AAAB01008849 was more precisely mapped in region 38C (Figure 2c).

The gene content and amounts of transposable elements and short simple repeats were compared between euchromatic and heterochromatic scaffolds across all chromosomes. Gene density in heterochromatin varies but, on average (2 per 100 kbp), is 40% that of the gene density in euchromatin (5 per 100 kbp). In the most centromeric scaffolds, gene content was as low as 0.2 per 100 kbp. The most significant components of the heterochromatic scaffolds are transposable elements: about 50% of the sequences were found to have similarities to the known transposable elements. The content of repeat elements shorter than 200 bp is two-fold higher in heterochromatic scaffolds (8.7%) than in euchromatic scaffolds (4.7%).

### Y-linked scaffolds

Recently, four satellite DNA families were reported from the male-specific Y chromosome [24]; however, the complete list of scaffolds harboring these satellite sequences was not published. In the present study, 54 such scaffolds have been identified using BLASTN searches. All 54 scaffolds are considered here as Y-linked (Additional data file 2). They usually have short sequences and are composed entirely of repeats of a given satellite family. The few exceptions correspond to scaffolds with juxtaposed arrays of different satellite families or of a satellite DNA and a transposable element fragment. Scaffolds containing Y-linked satellite DNA have a total length of 134 kb. Including the Y-linked scaffold detected previously [23], the overall length of the Y chromosome scaffolds identified in the *A. gambiae *genome reaches only 182 kb, making it still the most poorly explored part of the genome. None of the Y-specific scaffolds have been physically mapped, as *in situ *hybridization experiments were conducted only on polytene chromosomes from ovarian nurse cells.

### Assembly improvement in euchromatic regions

Analysis of BAC clones by *in situ *hybridization was used to assign four additional scaffolds (AAAB01008862, AAAB01008456, AAAB01008882, AAAB01008090) to the euchromatic chromosomal regions (Additional data file 1). In addition, scaffold AAAB01008838 was assigned to an inter-scaffold gap on 3L by *in situ *hybridization of cDNA clones. Scaffold AAAB01008882 was not included in the final 2L chromosome assembly because of the possibility of some miss-assembly.

A BLASTN analysis of BESs was utilized to identify 94 BAC clones that mapped in the vicinity of the 36 inter-scaffold gaps between scaffolds placed on chromosomes. These BAC clones were further examined manually to identify those that spanned gaps. On chromosome 2R, 13 BAC clones were identified that covered 11 gaps. Two BAC clones were identified on 2L that spanned one gap, two BAC clones on 3R covered two gaps and 19 BAC clones were identified on 3L that crossed a total of eight gaps. No BAC clones were identified that covered gaps on the X chromosome (Additional data file 3). In total, 36 BAC clones were identified that could be used to sequence through 22 gaps on the *Anopheles *genome assembly. As discussed below, 12 of these 22 gaps have also been bridged by finding that adjacent scaffold ends appear to represent alternative assemblies of the same region (Table 1).

### Detection of polymorphic and bacterial specific scaffolds

To identify scaffolds from polymorphic regions, unmapped scaffolds were aligned to the chromosome assemblies using the program exonerate [32], allowing alignments to extend through gaps and possible insertions and deletions in the scaffolds. This revealed 144 scaffolds, of sizes between 15 kbp and 415 kbp and totaling 8.186 Mbp, that aligned over their entire length to a previously mapped, larger scaffold (Additional data file 4). The two aligned alternatives for these regions differed in sequence by between 1.2% and 4.6%, with 90% of the pairs showing sequence differences within the range 1.7% to 3.7%. Such scaffolds probably represent alternative assemblies of the chromosome region and indicate parts of the genome where two haplotypes may have been segregating within the sequenced PEST strain. Seven scaffolds from this list were also physically mapped to appropriate chromosomal locations.

**Figure 3:**
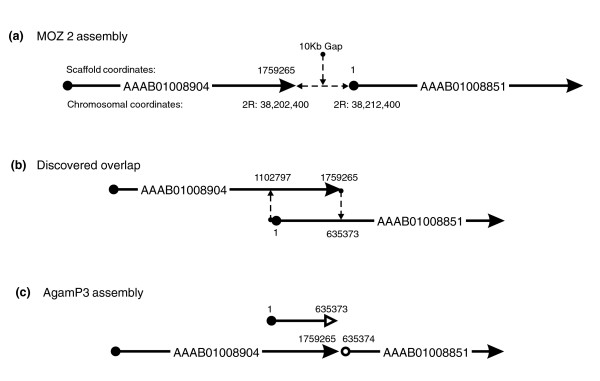
Example of joining scaffolds where adjacent ends are alternative assemblies of the same region. **(a) **Using physical mapping techniques, scaffolds AAAB01008904 and AAAB0108851 are placed adjacent to one another on chromosome arm 2R. In the previous genome assembly, MOZ2, the scaffolds were placed with an arbitrary 10 kbp of gap between them. **(b) **After alignment of scaffolds using Exonerate and Dotter, it was clear that there was about 64 kbp of sequence overlap between the 3' end of AAAB01008904 and the 5' end of AAAB0108851. Based on BAC coverage of each scaffold and gaps in each of the scaffold sequences, we chose to keep the overlapping region from AAAB01008904 (base-pairs 1102797 to 1759265) and use it for the new chromosome assembly. **(c) **The corresponding overlapping region from AAAB0108851 (base-pairs 1 to 635373) was deemed to be an alternative assembly segment, with the rest of the scaffold kept as part of the chromosome assembly. The regions retained as parts of chromosome arm 2R were placed adjacent to each other with no inter- scaffold gap.

Because such alternative assemblies could also occur at the ends of adjacent scaffolds, physically mapped scaffolds were also examined to detect ends that represented alternative assemblies of the same region. Two approaches were taken. First, all scaffolds were compared to all other scaffolds using exonerate, and long alignments of high identity that involved scaffold ends were examined. All such alignments detected involved pairs of scaffold ends that had been placed next to one another on the chromosome by physical mapping. Secondly, adjacent scaffold ends were aligned with Dotter, and the alignments were inspected visually. A final list of 21 scaffold segments on 18 scaffolds considered to be alternative assemblies was prepared by inspection of the exonerate and Dotter alignments (Additional data file 5). All these cases were in regions of chromosome arms 2R, 3L or 3R that were previously proposed to be segregating for distinct haplotypes in the PEST strain [1]. The range of sequence difference for the final set of aligned sequences was 2.0% to 4.0%. The segment with the higher quality sequence assembly, judged by number of gaps and separation of mapped BAC ends, was retained as part of the AgamP3 chromosome assembly. The lower quality segment was designated as an alternative assembly region (Figure 3). Approximately 3.53 Mbp of sequence were removed from the chromosomes by this approach and 20 gaps in the assembly were closed.

Initial analyses revealed that some of the unmapped scaffolds harbored sequences with unexpectedly high similarity to bacterial proteins. Thirty-two such scaffolds were tested for their presence in the mosquito genome by PCR amplification of *A. gambiae *PEST strain genomic DNA from embryos using PCR primer pairs specific to these scaffolds (Additional data file 6). Despite repeated attempts, none of the primer pairs yielded any specific products. The combined evidence of high sequence similarity to bacterial genes and negative PCR results strongly suggested that the bacterial-like sequences constitute a contaminant of the *A. gambiae *genome assembly, rather than an integral part of the *A. gambiae *genome (data not shown). To identify all such potential bacterial scaffolds, the entire unmapped scaffold set was compared against NCBI's nr protein database. Scaffolds were identified as bacterial contaminants if they had no high similarity to other *A. gambiae *scaffolds and top hits against the scaffold were only to bacterial proteins with E values at least five orders of magnitude higher than any hits to proteins from eukaryotic organisms. A set of 679 scaffolds, totaling 1.97 Mbp, matched these criteria and are thus regarded as bacterial (Additional data file 6).

The revised assembly (AgamP3) has a total of 80 scaffolds assigned to and ordered on the chromosome arms X, 2R, 2L, 3R and 3L (Table 1). The 28 scaffolds shown in bold have been newly mapped or oriented. In 10 cases, adjacent scaffolds are bridged by BAC clones that have their ends mapped to the two different scaffolds. In 20 cases adjacent scaffolds have been joined because their ends represent alternative assemblies of the same region; 12 of these joins are also supported by bridging BACs. Thus, three different approaches have proved valuable for improving the assembly of the genome: additional physical mapping, detailed *in silico *analysis of the scaffold sequences, and further mapping of BAC clone end sequences.

## Discussion

**Figure 4:**
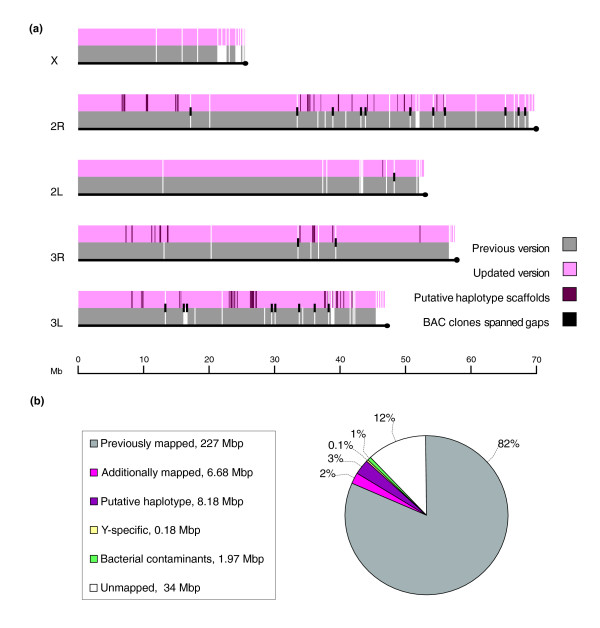
A comparison of the initial and updated versions of the *Anopheles gambiae *genome assembly.**(a) **The scaffolds from the previous and updated versions of the genome are shown by gray and pink bars, respectively. Purple stripes on the scaffolds indicate alternative haplotype scaffolds with sizes bigger than 50 kbp. Black bars correspond to the BAC clones that cross inter-scaffold gaps. **(b) **The updated status of the *A. gambiae *genome project. Sectors correspond to the previously mapped scaffolds, additionally physically mapped scaffolds, alternative haplotype scaffolds, Y-specific scaffolds, bacterial contaminant scaffolds and the remaining scaffolds that are not assigned to the chromosomes.

The result of this work is an improved view of the *A. gambiae *genome assembly. In the sequencing and assembly phase of the *A. gambiae *genome project, a significant amount of the heterochromatic DNA was successfully cloned and sequenced [1]. However, enrichment of the repetitive DNA in pericentromeric regions limited the initial effort to physically map these regions. Only 9 scaffolds with total size 3.3 Mbp were mapped in pericentromeric regions of chromosomes. Figure 4a compares this updated version of the *A. gambiae *assembly (AgamP3) with the previous version [1]. The most significant differences between these two versions are seen in the pericentromeric areas of all chromosomes. The updated version of the genome has 24 scaffolds with a total size of 8.64 Mbp in pericentromeric areas. These results are comparable with data obtained for the *D. melanogaster *genome. In the first publication of the fruit fly genome, only 3.8 Mbp were mapped to centromeric areas [20]. Release 3 of the *Drosophila *whole-genome shotgun sequence assembly (WGS3) significantly extended the assembly into the centric heterochromatin; 20.7 Mbp of sequence was identified as heterochromatic [33]. Both *Drosophila *and *Anopheles *genome assemblies have 16 large scaffolds with sizes bigger then 250 kbp in the heterochromatic regions of the chromosomes.

This AgamP3 assembly does not complete any of the centromeric regions on the chromosomes, and it is unclear if any of the scaffolds now mapped to centromeric regions actually include functional centromeric sequences. No large blocks of simple repeats appear in the scaffolds that have been mapped in heterochromatic regions. The amount of short repeats (smaller then 200 bp) in different heterochromatic scaffolds varies from 1% to 34%. The functional approximately 420 kbp *Drosophila *centromere is composed of large blocks of repeats (350 kbp) and more complex sequence composed of transposable elements [34]. The situation is similar in *Arabidopsis *chromosomes, where the centromeric regions contain tandem 180 bp repeats with a total size of about 0.5 to 3 Mbp, and the surrounding area is enriched in moderate repeats and transposable elements [16,17]. In neither case does the initial genome assembly reach the centromeric region, and special efforts were required for cloning and sequencing the centromeres.

According to *in situ *results, the only telomeric region covered by scaffolds in the *A. gambiae *assembly is on the 2L arm. All three satellite sequences previously described as telomeric [35,36] have been identified in this scaffold. The *in situ *results for the distal most BAC clones in the scaffolds closest to the telomeric regions on the other chromosomal arms showed that they are located several bands from the ends of the chromosomes.

The gene content in areas around centromeres is comparable between *Anopheles*, *Drosophila *and *Arabidopsis *genomes. Gene density in the *Anopheles *genome is 5 per 100 kbp in euchromatic scaffolds, 2 per 100 kbp in pericentromeric and 0.2 per 100 kbp in the three most centromeric scaffolds. In the *Drosophila *genome the gene content is higher in euchromatin at 11 genes per 100 kbp [20] and the same at 2 per 100 kbp around the heterochromatin-euchromatin junction [33]. *Arabidobsis *has an even higher gene content in euchromatic areas of about 25 per 100 kbp, 1.5 in the genetically identified centromeric region and 0.9 in the region enriched in repetitive elements [16]. As in *Drosophila *[37] and *Arabidopsis *[16], the *Anopheles *genome does not have a sharp boundary between hetero- and euchromatin.

Figure 4a shows 22 gaps between scaffolds in the *A. gambiae *genome that can be covered by additional sequencing of BAC clones, which would decrease the number of scaffolds in the genome assembly. The great progress in finishing the *Drosophila *genome has come as a result of the additional sequencing of overlapping BAC clones, sub-clones and PCR products [38]. Release 3 of the *Drosophila *genome is represented by 13 scaffolds with a total of 37 sequencing gaps in the euchromatic portion of the genome.

In the initial report of the *A. gambiae *genomic sequence, Holt *et al*. [1] described considerable genetic variation within the PEST strain and suggested, partly on the basis of finding regions with very high single nucleotide polymorphism (SNP) density, that the PEST strain continued to segregate into two different haplotypes for certain regions of the genome. These regions would have derived from the divergent Mopti and Savanna chromosomal forms that contributed to the construction of the PEST strain. Thomasova *et al*. [39] sequenced BAC clones in the *Pen1 *area of the PEST genome and found a 3.3% sequence difference in a 122 kbp region of BAC clone overlap, suggesting that this polymorphism in PEST was not simply an artifact of assembling a highly polymorphic colony. This study has identified 141 distinct scaffolds that probably represent alternative assemblies for regions totaling 8.2 Mbp, and an additional 3.5 Mbp previously mapped to chromosomes. Figure 4a shows the location of the alternative haplotype scaffolds with sizes bigger than 50 kbp. Adjacent pairs of scaffolds that have overlapping alternative assemblies on their ends are shown as single scaffolds on the picture of the new *A. gambiae *assembly.

It remains possible that some of the sequences designated as alternative haplotype assemblies are actually real duplications. However, the regions identified overlap with those previously found to have high SNP densities, and the alternative assemblies for a region differed in sequence by between 1.2% and 4.6%, similar to the previously reported difference found from BAC sequencing [39]. The identification of scaffolds that represent alternative assemblies enables duplicates to be removed from the set of scaffolds making up the genomic assembly and enables the elimination of artifactual genes from the predicted *A. gambiae *gene set. It will also facilitate initial studies of both non-coding and gene allelic differences between the two contributing chromosomal forms. It is important to note, however, that the two alternative assemblies of a region are unlikely to accurately represent the two alternative 'haplotypes' that may have been segregating in the PEST strain. Instead, the assembly process may produce two assemblies, both of which are a mosaic of the two haplotypes. Additional scaffolds or scaffold regions that represent alternative assemblies may still be present within the set described here as the revised genomic assembly AgamP3. In this study, 15 kbp was selected as the shortest alignment that could reliably be classified as two alternative assemblies, reasoning that smaller alignments could represent different transposons. In addition, some polymorphic regions may have been assembled as artifactual tandem duplications within a single scaffold [1]; this study has not attempted to eliminate such regions.

The *A. gambiae *genomic sequences are expected to contain some level of contamination from bacteria, particularly from those found in the gut [1]. Currently, 679 scaffolds have been identified as apparently bacterial. However, the actual number of bacterial scaffolds within the *A. gambiae *assembly may be larger. The selected list includes only scaffolds with BLAST hits to bacterial sequences having a cutoff value of E = 10^-15^. It is likely that some scaffolds with smaller sequence similarity to bacterial sequences currently available in GenBank also have bacterial origin.

Moreover, the assembly refinements described in this paper have a direct impact on the predicted genome wide set of genes. The most recent gene set based on the previous assembly [40] included 422 gene predictions on scaffolds or scaffold segments now classified as alternative assemblies. The scaffolds now designated as bacterial contaminants had 328 gene predictions already marked as of likely bacterial origin, and an additional 522 not so marked. Inspection of these showed that many had domains suggesting a likely bacterial origin, and none were unequivocally eukaryotic. Hence the first gene set based on the new assembly [41] benefits from the removal of some duplicate predictions (artifactual paralogues) for genes represented in two alternative assemblies of the same chromosome region and from the absence of predictions derived from bacterial contaminants.

## Conclusion

Use of cDNA and BAC clones and PCR amplified gene-fragments as probes for *in situ *hybridization and additional *in silico *analysis of the scaffold sequences have led to an overall improvement of the *A. gambiae *genome assembly. A total of about 15 Mbp has been added to the mapped part of the genome, about 2 Mbp has been removed as bacterial specific and about 12 Mbp has been reclassified as probable alternative assemblies (Figure 4b). One-third of the previously unmapped portion of the *A. gambiae *genome has been assigned to a chromosomal location. Removal of the probable bacterial and alternative assembly scaffolds has reduced the genome from the original total of 278 Mbp in the Moz2 assembly to 264 Mbp (without gaps), which is much closer to the C_o_T estimate of 260 Mbp of Besansky and Powell [21]. Moreover, even this new genome size estimate is likely to be somewhat inflated because of residual, small haplotype and bacterial contamination scaffolds. While the AgamP3 assembly can clearly be improved by sequencing BAC clones that cross inter-scaffold gaps or a careful analysis of mate pair violations among the genomic DNA clones sequenced in the original genome project, the upcoming genome projects for the *A. gambiae *S and M molecular forms [42] is almost certain to produce a significantly improved assembly for one or both of these two new *A. gambiae *genomes.

## Materials and methods

### In situ hybridization

Three different types of probe were used for *in situ *hybridization: cDNA clones, BAC clones and PCR amplified genes. The cDNA clones were selected from the ANGNAP1 library using BLASTN searching against each of the unmapped and unoriented scaffolds. A pair of cDNA clones from genes on opposite ends of the scaffold and each with a single location in the genome were considered as the candidates for *in situ *hybridization. BAC clones from ND-1 [1] or ND-Tam [26] libraries and PCR amplified gene-fragments were identified within the scaffolds using the mappings displayed in the VectorBase genome viewer [41]. Primer design for gene-fragment amplification was done using the Primer 3 program. The cDNA probes were prepared using the FastPlasmid Mini kit (Eppendorf, Hamburg, Germany) and BAC clone DNA was isolated by standard procedures [43]. Both types of probe were labeled with Cy3-AP3-dUTP or Cy5-AP3-dUTP (GE Healthcare UK Ltd (formerly Amersham Biosciences Corp.), Little Chalfont, Buckinghamshire, UK) using the GIBCO BRL Nick Translation labeling system. Amplified gene-fragment DNA was prepared by standard PCR amplification procedures [43]. To prevent non-specific amplification we utilized an appropriate BAC clone DNA as a template for PCR. PCR product was labeled with Cy3-AP3-dUTP or Cy5-AP3-dUTP (GE Healthcare UK Ltd) using the Random Primers DNA Labeling System (Invitrogen, Karlsruht, Germany). To obtain polytene chromosome preparations, ovaries from the SUA strain at the appropriate stage were dissected into fresh Carnoy's solution (ethanol: glacial acetic acid, 3:1). Ovaries were gently pressed with a cover slip in 50% propionic acid, dipped in liquid nitrogen, then cover slips were removed and slides were dehydrated in 50%, 70%, 95%, and 100% ethanol. DNA probes were hybridized to the chromosomes by standard procedures [44] and then chromosomes were washed in 0.2XSSC, counterstained with YOYO-1 and mounted in DABCO [45]. Fluorescent signals were detected using a Bio-Rad MRC 1024 Scanning Confocal System (Bio-Rad Laboratories, Hercules, CA, USA).

### Estimation of gene, transposable element and short repeat density in scaffolds

The genomic sequences of *A. gambiae *scaffolds were downloaded from the VectorBase website [41]. For the estimation of gene density in scaffolds, 12,600 assembled expressed sequence tags from a Normalized Head library, Normalized Fat Body library and a pooled library were placed on scaffolds using BLAST, requiring an E value of <10^-20^. For the analysis of the simple repeat content with sizes smaller then 200 bp, we used Tandem Repeats Finder [46]. The percentage of sequences corresponding to the known transposable elements was found using the RepeatMasker program [47]. Two custom databases were used for the search: the database of the *A. gambiae *transposable elements (Maria Sharakhova and Frank Collins, unpublished data) and the database of natural transposable element sequences identified in *D. melanogaster *by M Ashburner *et al*. [48].

### Identification of the Y-specific scaffolds

Scaffolds containing Y chromosome-specific satellite DNA families were regarded as Y-linked. They were identified using randomly selected monomer sequence of each of the four Y-specific satellite DNA families [24] as a query in local BLASTN searches against the *A. gambiae *genome database.

### Finding BAC clones bridging physical gaps between scaffolds

To identify BAC clones spanning gaps, BLASTN was utilized. *A. gambiae *BESs that demonstrated significant similarity (E < 10^-50^) to scaffolds on either side of gaps were selected. From this pool, BACs were identified as crossing gaps if paired BESs fulfilled the following criteria: matching the *A. gambiae *genomic sequence with an E value of <10^-75^, having the appropriate relative orientation, and preferably not being repeated on the scaffolds. The sequence length between the BES and the gap was then determined to identify the shortest BAC that crossed the gap, if more than one was identified, using the VectorBase genome viewer.

### Identification of polymorphic and bacterial contaminant scaffolds

To detect polymorphic scaffolds in the *A. gambiae *genome, unmapped scaffolds greater than 15 kbp in length were mapped to the release 2 chromosome assemblies using the program exonerate [32]. Alignment was carried out with exonerate's non-equivalence region (NER) model, which permits alignments to be carried on across the gaps within scaffolds and across possible insertions and deletions. Scaffolds were identified as representing putative alternative assemblies for a region if >97% of the unmapped scaffold was aligned to a chromosome region (where this figure includes any segments treated as non-equivalence regions) and there was >95% sequence identity in the aligned segments. A similar approach was used to identify ends of mapped scaffolds that might represent alternative assemblies of the same region. Selected scaffolds were also aligned and examined using Dotter [49]. Scaffolds were identified as derived from contaminating bacterial DNA if they were unmapped and if the scaffolds appeared to encode only proteins of prokaryotic origin. Scaffold sequences were compared with all proteins in GenBank using BLASTX and were designated as bacterial if they had a hit to a prokaryotic protein with an E value that was <10^-15 ^and 5 orders of magnitude lower than that of the best hit to a eukaryotic protein. To assess the risk of false positive results, we took scaffolds previously mapped to the *A. gambiae *chromosome arms, broke them into pieces of size equal to the average length of all putative contaminant scaffolds, and then searched them for prokaryotic-like proteins in the same manner. None of the mapped scaffolds would have been designated as bacterial by this procedure.

## Additional data files

The following additional data are available with the online version of this paper. Additional data file 1 contains a table listing scaffolds mapped to chromosomes. Additional data file 2 contains a table listing Y chromosome scaffolds. Additional data file 3 contains a table listing BAC clones that span scaffold gaps. Additional data file 4 contains a table containing a list of alternative assemblies. Additional data file 5 contains a table that lists segments of joined scaffolds that represent alternative assemblies of adjacent mapped scaffolds. Additional data file 6 contains a table that lists bacterial specific scaffolds.

## Supplementary Material

Additional data file 1 Scaffold AAAB01008882 was not included in the final 2L chromosome assembly because of concerns about possible mis-assembly of part of the scaffold. Click here for file

Additional data file 2 Scaffolds mapped to the Y chromosome. Click here for file

Additional data file 3 BAC clones that span gaps between scaffolds. Click here for file

Additional data file 4 Scaffolds representing alternative assemblies. Click here for file

Additional data file 5 Chr: the chromosome that the scaffolds are mapped to. Alt_Scaffold: the scaffold with sequence overlap considered to be an alternative assembly to the golden path. Chr_Scaffold: the scaffold with sequence overlap used for the golden path. as_start: start of the overlap on the scaffold with the alternative assembly segment (Alt_Scaffold). as_end: end of the overlap on the Alt_Scaffold. cs_start: start of the overlap on the scaffold where the overlap segment is used for the golden path (Chr_Scaffold). cs_end: end of the overlap on the Chr_Scaffold. Chr_Start: start of the overlap region, in chromosomal coordinates. Chr_End: end of the overlap region, in chromosomal coordinates. as_to_cs: orientation of the Alt_Scaffold with respect to the Chr_Scaffold. as_to_chr: orientation of the Alt_Scaffold with respect to the chromosome. Click here for file

Additional data file 6 Bacterial specific scaffolds. Click here for file

## Figures and Tables

**Table 1:** Scaffolds from the current *Anopheles gambiae *genome golden path

No.	Scaffold accession number	Full length of scaffold*	Scaffold begin	Scaffold end	Assembly status to the next scaffold	BAC clones crossing gap between current and next scaffold
	X chromosome	Telomere end				
1	AAAB01008846	11308833	4C	1D	Not joined	
2	AAAB01008847	3715079	1D	5A	Not joined	
3	AAAB01008963	2230633	5A	5C	Not joined	
4	AAAB01008811	3062431	5C	6	Not joined	
5	**AAAB01008973**	600295	6	6	Not joined	
6	**AAAB01008958**	589940	6	6	Not joined	
7	AAAB01008852	409660	6	6	Not joined	
8	**AAAB01008975**	935344	6	6	Not joined	
9	**AAAB01008885**	267815	6	6	Not joined	
10	**AAAB01008967**	438965	6	6	Not joined	
11	AAAB01007622	14705	6	6	Not joined	
12	**AAAB01008976**	589797	6	6	Not joined	
13	**AAAB01008861**	109611	6	6	Not joined	
	X chromosome	Centromere end				
	2R chromosome	Telomere end				
14	AAAB01008987	16222597	10D	7A	Bridged	17O20
15	AAAB01008799	2774677	11B	10D	Not joined	
16	AAAB01008859	12516315	13E	11B	Bridged	122O11
17	AAAB01008879	2921310	14A	14C	Joined	
18	AAAB01008794	932688	14D	14D	Joined	
19	AAAB01008982	1015562	14D	14E	Joined and bridged	155O10
20	AAAB01008904	1759265	14E	15B	Joined	
21	AAAB01008851	2082253	15C	15C	Joined and bridged	179J17
22	AAAB01008820	590116	15D	15C	Joined and bridged	30L16
23	AAAB01008888	3396474	15D	16A	Not joined	
24	AAAB01008844	2866027	16B	16D	Joined and bridged	21H06
25	AAAB01008805	646796	16D	16D	Not joined	
26	**AAAB01008862**	212521	16D	16D	Not joined	
27	AAAB01008978	1934381	16D	17B	Bridged	124P12
28	AAAB01008817	1590424	17C	17C	Bridged	16N20, 105N12
29	AAAB01008880	4233641	18A	18C	Not joined	
30	AAAB01008898	4120773	18C	19C	Bridged	105P15
31	**AAAB01008952**	1118246	19D	19C	Not joined	
32	**AAAB01008961**	516376	19D	19D	Bridged	174H20, 127O12
33	**AAAB01008850**	840256	19D	19D	Bridged	07F16
34	**AAAB01008977**	457753	19D	19D	Not joined	
35	**AAAB01008949**	335163	19E	19E	Not joined	
36	**AAAB01008897**	259841	19E	19E	Not joined	
	2R chromosome	Centromere end				
	2L chromosome	Centromere end				
37	**AAAB01008942**	373146	20A	20A	Not joined	
38	**AAAB01008026**	124951	20A	20A	Not joined	
39	AAAB01008864	318965	20B	20B	Not joined	
40	AAAB01008968	3184012	20D	20B	Bridged	01I16, 01J12
41	AAAB01008905	1027887	20D	20D	Not joined	
42	AAAB01008948	3345744	21A	21B	Not joined	
43	**AAAB01008456**	41134	21B	21B	Not joined	
44	**AAAB01008827**	28099	21B	21B	Not joined	
45	AAAB01008900	4906461	21C	21F	Not joined	
46	AAAB01008810	494023	21F	22A	Not joined	
47	AAAB01008960	23099915	22A	25D	Not joined	
48	AAAB01008807	12309988	28D	25D	Not joined	
	2Lchromosome	Telomere end				
	3R chromosome	Telomere end				
49	AAAB01008964	12399987	30E	29A	Joined	
50	AAAB01008944	6709423	30E	31D	Joined	
51	AAAB01008984	12483120	32A	33D	Joined and bridged	11E04
52	AAAB01008835	1771096	33D	34A	Joined	
53	AAAB01008797	1002333	34A	34B	Not joined	
54	AAAB01008839	2408169	34C	34B	Joined and bridged	08B11
55	AAAB01008980	16417966	34C	37D	Not joined	
56	**AAAB01008822**	56627	37D	37D	Not joined	
57	**AAAB01008971**	377729	37D	37D	Not joined	
58	**AAAB01008943**	173243	37D	37D	Not joined	
	3R chromosome	Centromere end				
	3L chromosome	Centromere end				
59	**AAAB01008981**	219224	38A	38A	Not joined	
60	**AAAB01008985**	236235	38A	38A	Not joined	
61	**AAAB01008972**	744379	38A	38A	Not joined	
62	**AAAB01008957**	222192	38A	38A	Not joined	
63	AAAB01008849	2994010	38C	38B	Not joined	
64	AAAB01008906	127247	38C	38C	Not joined	
65	**AAAB01008795**	347814	38C	38C	Not joined	
66	AAAB01008933	2255294	38C	39B	Not joined	
67	**AAAB01008838**	221210	39A	39A	Not joined	
68	AAAB01008796	270581	39B	39B	Bridged	22E23, 119N12
69	AAAB01008848	1927899	39B	39C	Joined and bridged	133F8, 12F24
70	AAAB01008979	1577277	40A	39C	Joined	
71	AAAB01008951	359421	40A	40A	Joined and bridged	02H06
72	AAAB01008823	3392972	41A	40B	Joined and bridged	04P06, 10C06, 08F18
73	AAAB01008793	402616	41A	41A	Joined and bridged	160H13
74	AAAB01008804	907607	41B	41A	Joined	
75	AAAB01008816	6058108	42B	41B	Not joined	
76	AAAB01008966	3863510	43B	42C	Joined	
77	AAAB01008956	1048260	43B	43B	Joined and bridged	128N23
78	**AAAB01008090**	381451	43B	43B	Joined and bridged	131N20, 143K17, 23C10
79	AAAB01008834	2541584	43D	43B	Bridged	12N18, 08K01, 102F22, 172A24, 19C24, 23K03
80	AAAB01008986	12698247	46D	43D	Not joined	
	3L chromosome	Telomere end				

*This represents the original scaffold lengths. When adjacent scaffolds overlap, part of one of the scaffolds was designated as an alternative assembly and excluded from the chromosome assembly (see Additional data file 6). The 28 scaffolds shown in bold have been newly mapped or oriented.
