# Supplementary material for: Update of the Anopheles gambiae PEST genome assembly
Source: Genome Biol. 2007 Jan 8;8(1):R5. doi: 10.1186/gb-2007-8-1-r5 (PMC1839121; doi:10.1186/gb-2007-8-1-r5)
Supplement: Additional data file 2 — Scaffolds mapped to the Y chromosome. [file gb-2007-8-1-r5-S2.doc]

AAAB01001191, AAAB01001256, AAAB01002719, AAAB01002919, AAAB01003000, AAAB01003029, AAAB01003170, AAAB01003172, AAAB01003179, AAAB01003212, AAAB01003250, AAAB01003305, AAAB01003360, AAAB01003590, AAAB01003591, AAAB01003610, AAAB01003738, AAAB01003818, AAAB01003828, AAAB01003882, AAAB01003901, AAAB01003918, AAAB01003936, AAAB01004029, AAAB01004059, AAAB01004063, AAAB01004140, AAAB01004290, AAAB01004300, AAAB01004336, AAAB01004339, AAAB01004374, AAAB01004453, AAAB01004515, AAAB01004621, AAAB01004651, AAAB01004779, AAAB01004782, AAAB01005036, AAAB01005040, AAAB01005176, AAAB01005257, AAAB01005283, AAAB01005354, AAAB01005560, AAAB01006901, AAAB01006906, AAAB01006929, AAAB01006979, AAAB01007001, AAAB01007180, AAAB01007230, AAAB01007761, AAAB01007832, AAAB01008227
