# Supplementary material for: Update of the Anopheles gambiae PEST genome assembly
Source: Genome Biol. 2007 Jan 8;8(1):R5. doi: 10.1186/gb-2007-8-1-r5 (PMC1839121; doi:10.1186/gb-2007-8-1-r5)
Supplement: Additional data file 6 — Bacterial specific scaffolds. [file gb-2007-8-1-r5-S6.doc]

Bacterial specific scaffolds. Scaffolds in bold were tested for the presence in the *A. gambiae* genome using PCR and yielded negative results.

AAAB01000630, AAAB01000655, AAAB01000700, AAAB01000710, **AAAB01000754,** AAAB01000756, AAAB01000772, AAAB01000805, AAAB01000814, AAAB01000855, AAAB01000877, AAAB01000883, AAAB01000899, AAAB01000901, AAAB01000911, AAAB01000943, AAAB01000952, AAAB01000962, AAAB01001002, AAAB01001027, AAAB01001031, AAAB01001077, AAAB01001085, **AAAB01001092,** AAAB01001100, **AAAB01001104,** AAAB01001109, AAAB01001116, AAAB01001198, AAAB01001250, AAAB01001315, AAAB01001339, AAAB01001356, AAAB01001366, AAAB01001384, AAAB01001461, AAAB01001473, AAAB01001477, AAAB01001481, AAAB01001530, AAAB01001532, AAAB01001534, AAAB01001582, AAAB01001600, AAAB01001604, AAAB01001615, AAAB01001619, AAAB01001683, AAAB01001691, AAAB01001708, AAAB01001730, AAAB01001761, AAAB01001842, AAAB01001848, AAAB01001863, AAAB01001866, AAAB01001894, AAAB01001905, AAAB01001916, AAAB01001918, AAAB01001923, AAAB01001961, AAAB01001970, AAAB01001985, AAAB01001999, AAAB01002048, AAAB01002072, AAAB01002097, AAAB01002104, AAAB01002109, AAAB01002134, AAAB01002174, AAAB01002193, AAAB01002263, AAAB01002294, AAAB01002363, AAAB01002406, AAAB01002421, AAAB01002482, AAAB01002485, AAAB01002517, AAAB01002544, AAAB01002556, AAAB01002592, AAAB01002609, AAAB01002615, AAAB01002617, AAAB01002623, AAAB01002627, AAAB01002629, AAAB01002635, AAAB01002642, AAAB01002648, AAAB01002649, AAAB01002656, **AAAB01002668,** AAAB01002669, AAAB01002672, AAAB01002673, **AAAB01002674,** AAAB01002681, AAAB01002682, AAAB01002686, AAAB01002691, AAAB01002692, AAAB01002704, AAAB01002707, AAAB01002709, AAAB01002714, AAAB01002715, AAAB01002717, AAAB01002720, AAAB01002722, AAAB01002724, AAAB01002726, AAAB01002730, AAAB01002732, AAAB01002736, AAAB01002737, AAAB01002745, AAAB01002746, AAAB01002747, AAAB01002748, AAAB01002750, AAAB01002751, AAAB01002758, AAAB01002759, AAAB01002766, AAAB01002767, AAAB01002775, AAAB01002779, AAAB01002785, AAAB01002786, AAAB01002789, AAAB01002793, AAAB01002794, AAAB01002796, AAAB01002805, AAAB01002815, AAAB01002817, AAAB01002824, AAAB01002825, AAAB01002826, AAAB01002830, AAAB01002841, AAAB01002842, AAAB01002844, AAAB01002845, AAAB01002846, AAAB01002848, AAAB01002849, AAAB01002852, AAAB01002854, AAAB01002858, AAAB01002861, AAAB01002865, AAAB01002870, AAAB01002871, AAAB01002873, AAAB01002875, AAAB01002881, AAAB01002891, AAAB01002892, AAAB01002897, AAAB01002898, AAAB01002899, AAAB01002901, AAAB01002904, AAAB01002906, **AAAB01002914,** AAAB01002915, AAAB01002916, AAAB01002920, AAAB01002927, AAAB01002943, AAAB01002946, AAAB01002948, AAAB01002950, AAAB01002952, AAAB01002953, AAAB01002960, AAAB01002966, AAAB01002970, **AAAB01002978,** AAAB01002980, AAAB01002982, AAAB01002987, AAAB01002993, AAAB01002997, AAAB01003003, AAAB01003006, AAAB01003008, AAAB01003011, AAAB01003012, AAAB01003015, AAAB01003020, AAAB01003023, AAAB01003034, AAAB01003039, AAAB01003041, AAAB01003042, **AAAB01003043,** AAAB01003046, AAAB01003049, AAAB01003051, AAAB01003055, AAAB01003057, AAAB01003058, AAAB01003062, AAAB01003067, AAAB01003069, AAAB01003072, AAAB01003081, AAAB01003088, AAAB01003090, AAAB01003092, AAAB01003096, AAAB01003104, AAAB01003106, AAAB01003109, **AAAB01003112,** AAAB01003122, AAAB01003123, AAAB01003134, AAAB01003136, AAAB01003137, AAAB01003142, AAAB01003143, AAAB01003145, AAAB01003149, **AAAB01003151,** AAAB01003152, AAAB01003165, AAAB01003167, **AAAB01003173,** AAAB01003174, AAAB01003180, AAAB01003182, AAAB01003183, **AAAB01003189,** AAAB01003191, AAAB01003196, AAAB01003197, AAAB01003207, AAAB01003209, **AAAB01003220,** AAAB01003222, AAAB01003240, **AAAB01003260,** AAAB01003266, AAAB01003273, AAAB01003286, AAAB01003288, AAAB01003296, AAAB01003302, AAAB01003307, AAAB01003309, AAAB01003312, AAAB01003319, AAAB01003322, AAAB01003327, AAAB01003332, AAAB01003333, AAAB01003334, AAAB01003335, AAAB01003336, AAAB01003350, AAAB01003362, AAAB01003363, **AAAB01003368,** AAAB01003370, AAAB01003372, AAAB01003377, AAAB01003379, AAAB01003386, AAAB01003387, AAAB01003388, AAAB01003393, AAAB01003401, AAAB01003407, AAAB01003411, AAAB01003415, AAAB01003419, AAAB01003422, AAAB01003425, AAAB01003427, AAAB01003431, AAAB01003433, AAAB01003434, **AAAB01003436,** AAAB01003437, AAAB01003440, AAAB01003446, AAAB01003447, AAAB01003455, AAAB01003456, AAAB01003463, AAAB01003466, AAAB01003469, AAAB01003475, AAAB01003476, AAAB01003478, AAAB01003479, AAAB01003481, AAAB01003482, AAAB01003490, AAAB01003491, AAAB01003492, AAAB01003494, AAAB01003503, AAAB01003505, AAAB01003506, AAAB01003522, AAAB01003527, AAAB01003536, AAAB01003546, AAAB01003547, AAAB01003548, AAAB01003555, AAAB01003558, AAAB01003560, AAAB01003561, AAAB01003565, AAAB01003566, AAAB01003569, AAAB01003574, AAAB01003580, AAAB01003583, AAAB01003586, AAAB01003588, AAAB01003592, AAAB01003594, AAAB01003598, AAAB01003600, AAAB01003603, AAAB01003605, AAAB01003606, AAAB01003611, AAAB01003615, AAAB01003629, AAAB01003631, AAAB01003632, AAAB01003633, AAAB01003640, AAAB01003644, AAAB01003653, AAAB01003658, AAAB01003688, AAAB01003692, AAAB01003707, AAAB01003708, AAAB01003713, AAAB01003716, **AAAB01003737,** AAAB01003747, AAAB01003754, AAAB01003757, AAAB01003770, AAAB01003771, AAAB01003772, AAAB01003783, AAAB01003837, AAAB01003840, AAAB01003841, AAAB01003844, AAAB01003861, AAAB01003862, AAAB01003865, **AAAB01003872,** AAAB01003875, AAAB01003876, AAAB01003887, AAAB01003896, AAAB01003920, AAAB01003924, AAAB01003925, AAAB01003926, AAAB01003929, AAAB01003931, AAAB01003943, AAAB01003948, AAAB01003955, AAAB01003971, AAAB01003973, AAAB01003987, AAAB01003988, AAAB01003999, AAAB01004000, AAAB01004004, AAAB01004012, AAAB01004014, AAAB01004027, AAAB01004030, AAAB01004034, AAAB01004040, AAAB01004041, AAAB01004055, AAAB01004066, AAAB01004076, AAAB01004099, AAAB01004123, AAAB01004143, AAAB01004152, AAAB01004165, AAAB01004168, AAAB01004179, AAAB01004180, AAAB01004189, AAAB01004192, AAAB01004193, AAAB01004200, AAAB01004221, AAAB01004234, AAAB01004286, AAAB01004294, AAAB01004311, AAAB01004328, AAAB01004330, AAAB01004353, AAAB01004373, AAAB01004380, AAAB01004401, AAAB01004416, AAAB01004428, AAAB01004439, AAAB01004443, AAAB01004461, AAAB01004467, AAAB01004471, AAAB01004499, AAAB01004524, AAAB01004545, AAAB01004548, AAAB01004549, AAAB01004552, AAAB01004554, AAAB01004561, **AAAB01004569,** AAAB01004573, AAAB01004574, AAAB01004582, AAAB01004595, AAAB01004630, AAAB01004643, AAAB01004650, AAAB01004652, AAAB01004666, **AAAB01004682,** AAAB01004715, **AAAB01004726,** AAAB01004729, AAAB01004759, **AAAB01004767,** AAAB01004768, **AAAB01004772,** AAAB01004780, AAAB01004790, AAAB01004792, AAAB01004809, AAAB01004810, AAAB01004825, AAAB01004883, AAAB01004896, AAAB01004914, AAAB01004924, AAAB01004956, AAAB01004957, AAAB01004963, AAAB01004968, AAAB01004979, AAAB01004994, AAAB01005018, AAAB01005042, AAAB01005068, AAAB01005070, AAAB01005079, **AAAB01005098,** AAAB01005109, AAAB01005112, AAAB01005118, AAAB01005135, AAAB01005143, AAAB01005156, AAAB01005168, AAAB01005174, AAAB01005196, AAAB01005198, AAAB01005201, AAAB01005210, AAAB01005219, AAAB01005224, AAAB01005245, AAAB01005250, AAAB01005272, AAAB01005275, AAAB01005305, AAAB01005311, AAAB01005319, AAAB01005331, AAAB01005346, AAAB01005393, AAAB01005396, AAAB01005400, AAAB01005407, AAAB01005414, AAAB01005425, AAAB01005457, AAAB01005461, AAAB01005498, AAAB01005500, AAAB01005517, AAAB01005540, AAAB01005558, AAAB01005577, AAAB01005636, AAAB01005644, AAAB01005655, AAAB01005667, AAAB01005677, AAAB01005678, AAAB01005679, AAAB01005721, AAAB01005726, AAAB01005750, AAAB01005759, AAAB01005775, AAAB01005781, AAAB01005786, AAAB01005792, AAAB01005805, AAAB01005812, AAAB01005824, AAAB01005851, AAAB01005855, AAAB01005856, AAAB01005862, AAAB01005873, AAAB01005876, AAAB01005898, AAAB01005909, AAAB01005917, AAAB01005932, AAAB01005956, AAAB01005976, AAAB01006020, AAAB01006024, AAAB01006028, AAAB01006066, AAAB01006074, AAAB01006083, AAAB01006093, AAAB01006097, AAAB01006105, AAAB01006107, AAAB01006110, AAAB01006113, AAAB01006114, AAAB01006115, AAAB01006131, AAAB01006149, AAAB01006167, AAAB01006171, AAAB01006173, AAAB01006174, AAAB01006179, AAAB01006180, AAAB01006181, AAAB01006183, AAAB01006188, AAAB01006189, AAAB01006190, AAAB01006192, AAAB01006193, AAAB01006194, AAAB01006195, AAAB01006197, AAAB01006202, AAAB01006203, AAAB01006205, AAAB01006207, AAAB01006208, AAAB01006209, AAAB01006210, AAAB01006211, AAAB01006212, AAAB01006213, AAAB01006214, AAAB01006215, AAAB01006217, AAAB01006221, AAAB01006227, AAAB01006228, AAAB01006231, AAAB01006236, AAAB01006237, AAAB01006238, AAAB01006242, AAAB01006244, AAAB01006245, AAAB01006247, AAAB01006249, AAAB01006251, AAAB01006253, AAAB01006255, AAAB01006256, AAAB01006257, AAAB01006258, AAAB01006261, AAAB01006262, AAAB01006264, AAAB01006265, AAAB01006267, AAAB01006271, AAAB01006272, AAAB01006273, AAAB01006274, AAAB01006275, AAAB01006276, AAAB01006281, AAAB01006282, AAAB01006284, AAAB01006285, AAAB01006287, AAAB01006290, AAAB01006294, AAAB01006295, AAAB01006298, AAAB01006300, AAAB01006305, AAAB01006309, AAAB01006310, AAAB01006311, AAAB01006312, AAAB01006315, AAAB01006316, AAAB01006318, AAAB01006322, AAAB01006323, AAAB01006324, AAAB01006329, AAAB01006332, AAAB01006336, AAAB01006347, AAAB01006356, AAAB01006358, AAAB01006360, AAAB01006381, AAAB01006463, AAAB01006473, AAAB01006481, AAAB01006520, AAAB01006522, AAAB01006889, AAAB01006965, AAAB01007005, AAAB01007030, AAAB01007040, **AAAB01007134,** AAAB01007250, AAAB01007357, AAAB01007364, AAAB01007394, AAAB01007395, AAAB01007397, AAAB01007585, AAAB01007601, AAAB01007830, AAAB01007848, AAAB01007849, AAAB01008631, AAAB01008714, AAAB01008715, **AAAB01008716,** AAAB01008717, AAAB01008718, AAAB01008728, AAAB01008730, AAAB01008739, AAAB01008740
